# Increased body weight in mice with *fragile X messenger ribonucleoprotein 1* (*Fmr1*) gene mutation is associated with hypothalamic dysfunction

**DOI:** 10.1038/s41598-023-39643-z

**Published:** 2023-08-04

**Authors:** Rebecca E. Ruggiero-Ruff, Pedro A. Villa, Sarah Abu Hijleh, Bryant Avalos, Nicholas V. DiPatrizio, Sachiko Haga-Yamanaka, Djurdjica Coss

**Affiliations:** 1 Division of Biomedical Sciences, School of Medicine, University of California, Riverside, Riverside, CA 92521 USA; 2 Department of Molecular, Cell, and Systems Biology, College of Natural and Agricultural Sciences, University of California, Riverside, Riverside, USA

**Keywords:** Developmental disorders, Cellular neuroscience, Feeding behaviour, Hypothalamus, Obesity

## Abstract

Mutations in the *Fragile X Messenger Ribonucleoprotein 1* (*FMR1*) gene are linked to Fragile X Syndrome, the most common monogenic cause of intellectual disability and autism. People affected with mutations in *FMR1* have higher incidence of obesity, but the mechanisms are largely unknown. In the current study, we determined that male *Fmr1* knockout mice (KO, *Fmr1*^*−/y*^), but not female *Fmr1*^−/−^, exhibit increased weight when compared to wild-type controls, similarly to humans with *FMR1* mutations. No differences in food or water intake were found between groups; however, male *Fmr1*^*−/y*^ display lower locomotor activity, especially during their active phase. Moreover, *Fmr1*^*−/y*^ have olfactory dysfunction determined by buried food test, although they exhibit increased compulsive behavior, determined by marble burying test. Since olfactory brain regions communicate with hypothalamic regions that regulate food intake, including POMC neurons that also regulate locomotion, we examined POMC neuron innervation and numbers in *Fmr1*^*−/y*^ mice. POMC neurons express Fmrp, and POMC neurons in *Fmr1*^*−/y*^ have higher inhibitory GABAergic synaptic inputs. Consistent with increased inhibitory innervation, POMC neurons in the *Fmr1*^*−/y*^ mice exhibit lower activity, based on cFOS expression. Notably, *Fmr1*^*−/y*^ mice have fewer POMC neurons than controls, specifically in the rostral arcuate nucleus, which could contribute to decreased locomotion and increased body weight. These results suggest a role for *Fmr1* in the regulation of POMC neuron function and the etiology of *Fmr1*-linked obesity.

## Introduction

Mutations in the *Fragile X Messenger Ribonucleoprotein 1* (*FMR1*) gene cause Fragile X Syndrome (FXS), the most common genetic form of intellectual disability^1,2^. People affected with this disorder have mental impairment, autism and higher incidence of obesity^3–7^. Mutation entails expansion of the unstable CGG trinucleotide repeats, which leads to hypermethylation, silencing of the gene and the loss of protein product, FMRP. FMRP is an mRNA binding protein that regulates protein levels of its targets^8,9^. In the brain, where it is most highly expressed, FMRP binds mRNAs that encode synaptic proteins, contributing to cognitive dysfunctions in FXS^10–12^. While the mechanisms of intellectual impairments following FMRP loss are beginning to emerge, mechanisms of increased weight are not known. The effect of FMRP loss on the cortex and hippocampus have been analyzed^13,14^, however, how mutations affect hypothalamic functions has not been examined. Herein, we investigated the effects of FMRP loss in the regulation of body weight and food intake, using the *Fmr1* knock-out (*Fmr1*^*−/y*^, KO) mouse model. Due to differential methylation between human and mouse genes, the *Fmr1* KO is a widely used mouse model to study Fragile X Syndrome and is considered a better model than putative mimics of the CGG repeat expansion^15,16^. We analyzed effects of the lack of Fmrp on food intake and specifically on a population of hypothalamic neurons that regulate feeding, satiety and energy expenditure.

*FMR1* mutations are associated with increased obesity particularly in children. 34% of pediatric patients with FXS experience obesity compared to 18% of unaffected children^3–6^. Obesity, especially in childhood, leads to an increased risk of cardiovascular disease, metabolic syndrome, dementia, and stroke. The causes of increased obesity in FXS are not clear. Food intake and energy expenditure are regulated by the hypothalamus, which also controls other homeostatic processes, such as circadian rhythms, thermoregulation, stress response, and reproductive function. Our previous study analyzed the role of the *Fmr1* gene in reproduction, since women with *FMR1* mutation experience early cessation of reproductive function and males have macroorchidism^17,18^. We demonstrated increased innervation of ovarian follicles and of GnRH neurons in the hypothalamus that regulate reproduction^19^. The hypothalamus receives information on availability of energy stores from the periphery to regulate food intake^20,21^. Metabolic cues are integrated primarily by anorexigenic proopiomelanocortin (POMC) neurons and orexigenic neuropeptide Y (NPY) / agouti-related protein (AgRP) neurons located in the arcuate nucleus (ARC) of the mediobasal hypothalamus^22^. AgRP neurons in the sated state lower GABA tone that normally inhibits POMC neurons^23,24^. This disinhibition leads to activation of POMC neurons and synthesis of the POMC peptide. POMC is cleaved to several neuropeptides, most importantly to alpha-melanocyte stimulating hormones (αMSH) that plays a role in weight regulation by binding to melanocortin 4 receptor (MC4R). Signaling through this receptor, located in several nuclei, including paraventricular nucleus (PVN), ventromedial hypothalamus (VMH) and brainstem, in the brain helps maintain the balance between food intake and energy expenditure^25–27^. Given that *FMR1* gene mutations are associated with increased obesity, it is critical to examine *FMR1* role in the regulation of energy balance.

Individuals with FXS exhibit abnormal sensory information processing leading to hypersensitivity to a variety to sensory inputs, which results in a wide array of behavioral symptoms^13^. This may be a result of alteration in several neurotransmitter receptor levels resulting in decreased inhibitory/excitatory synaptic balance and the diminished synaptic plasticity^28^. Mutations of the *FMR1* gene may affect olfaction, but the results are not clear. *Fmr1* KO mice have decreased ability to detect smell, but no differences from controls in distinguishing between different odorants^29^. Olfaction in animals is critical for food detection and intake, and the olfactory bulb projects to the hypothalamus and feeding circuitry to stimulate hunger and food intake^30,31^. However, exact mechanisms and pathways that connect olfactory brain regions with the hypothalamus to stimulate hunger, or to the regions that regulate locomotion to increase foraging for food, are not clear. In this study, we analyzed feeding and energy expenditure in *Fmr1* KO mice, and hypothalamic neurons that regulate these processes. We determined that male KO mice are heavier than controls, but lack differences in food intake, while locomotion was affected. We identified profound effects on olfaction and POMC neurons that regulate energy expenditure. Therefore, olfaction and alterations in activity of POMC neurons likely contribute to the dysregulation of energy balance and increased obesity in people affected with *FMR1* mutations.

## Materials and methods

### Animals

All animal procedures were performed with the approval from the University of California (Riverside, CA) Animal Care and Use Committee and in agreement with the National Institutes of Health Animal care and Use Guidelines. The study was performed, and results reported in accordance to ARRIVE guidelines. Breeding pairs of FVB.129P2-Fmr1tm1Cgr*/J* (*Fmr1* KO) and their congenic controls (WT) mice were obtained from Jackson Laboratories and bred in-house. Mice were maintained under a 12-h light, 12-h dark cycle and received food and water ad libitum. *Fmr1* KO mice have larger litters^19^ and to prevent litter size influence on pup weight, litter sizes were normalized to 8 mice per litter. Previous studies using FXS mouse models demonstrated that these mice have heightened response to stress and altered levels of glucocorticoids^32^. To reduce stress, animals were acclimated by daily handling.

### Feeding behavior and locomotor activity

WT and *Fmr1* KO mice between 8 and 10 weeks of age were individually housed in Phenomaster feeding chambers (TSE Systems, Chesterfield, MO, USA) to analyze food intake and locomotion, and received access to standard chow and water ad libitum throughout behavioral testing. These chambers allow for continuous monitoring of food and water intake, and rigorous analysis of locomotor activity given that frames are equipped with light-beam grid and infrared sensors in three dimensions. Animals were allowed 72 h to acclimate, after which food intake, water intake, and locomotion were recorded. Food weight, water, and continuous locomotor activity were measured in real time and recorded using the TSE Phenomaster Software.

### Behavioral tests

#### Buried and unburied food test

Buried and unburied food tests were performed as previously described^33,34^. Male and female mice, 8–12 weeks of age, after 24-h fasting period, were used to locate buried or unburied standard chow pellet, starting at 7 pm, at the start of their active cycle and dark period in our colony. Polycarbonate cages with clean bedding material at a depth of 5 cm were used for testing. A single mouse was placed at the center of a test cage and allowed to acclimate for 5 min. After acclimatization, the mouse was placed in a clean holding cage, the pellet was buried, and the mouse was placed back in the test cage and allowed to recover the buried food pellet within a 10 min test time. Separate holding and test cages were used for each mouse. Latency, defined as the time between placing the mouse into the cage and the mouse grasping the food pellet with its forepaws, was recorded. Latency of animals that did not recover the food pellet within 10 min was recorded as 600 s. One week after the buried food test was performed, the mice underwent the unburied food test. The acclimation and testing conditions were identical to the buried food test, except the mice were timed in grasping the food pellet that was left on the top of bedding.

### Marble burying test

Marble burying test was performed as previously described^35^. Briefly, Male and female mice, 8–12 weeks of age were tested at 7 pm, as described above. Polycarbonate cages with fitted filter tops were filled with fresh unscented mouse bedding to a depth of 5 cm. Standard glass marbles were washed in mild laboratory detergent, rinsed with distilled-deionized water and dried, and then spaced out evenly in five rows of four marbles on the top of bedding. Test recording started immediately after the animal was placed in the cage, far away from the marbles as possible. The animals were left to explore undisturbed for 30 min. Marbles were counted and scored as buried if two-thirds of its surface was covered by bedding.

### Histological analyses and immunohistochemistry

WT controls and *Fmr1* KO mice were anesthetized, perfused with 20 ml PBS and 20 ml 4% paraformaldehyde; and tissues were collected. Hypothalami were sectioned to 50 μm coronal sections. Sections containing paraventricular nucleus (PVN) and arcuate nucleus (ARC) where MC4R or POMC neurons are located, respectively, were blocked and stained for β-endorphin for POMC neuron detection (1:10,000 dilution, rabbit anti-β-endorphin, Phoenix Pharmaceuticals H-022-33), MC4R (1:1000 dilution, rabbit anti-MC4R, Abcam ab24233), GABAγ2 receptor subunit (1:10,000 dilution, guinea pig anti-GABAγ2, Synaptic systems 224 004), VGAT (1:5000, mouse anti-VGAT, Synaptic systems 131 011) or cFOS (1:10,000, guinea pig anti-cFOS, Synaptic systems 226 308) for 48 h at 4 °C. After PBST washes, sections were incubated overnight at 4 °C with secondary antibodies: goat anti-rabbit IgG-Alexa Fluor 488 (1:5000, Invitrogen, A11034); anti-mouse IgG-Alexa Fluor 594 (1:1000, A11032, Invitrogen); anti-guinea pig–biotin (1:1000, BA-7000, Vector Laboratories, Burlingame, CA) followed by streptavidin-Cy5 (1:1000, 434316, Vector Laboratories, Burlingame, CA). Secondary antibody-only controls were performed to determine antibody specificity. MC4R neurons in the PVN were quantified by mean fluorescent intensity (MFI) using Fiji ImageJ. Numbers of POMC neurons were quantified by counting β-endorphin/POMC stained cell bodies in the coronal sections throughout the arcuate nucleus of the hypothalamus. To quantify the number of cFOS-expressing POMC neurons, 50 μm coronal sections of the arcuate nucleus were stained for POMC and cFOS, and results represented as a percentage of cFOS-positive POMC neurons. Counts were recorded by an investigator blinded to the group, which was revealed after counts were obtained. All sections were imaged using Leica microscope (DM6000) and analyzed using Fiji ImageJ.

Immunostaining for FMRP was performed using free-floating 50 μm sections spanning PVN (for MC4R-FMRP) or ARC (POMC/β-endorphin-FMRP). Sections were stained for 48 h with mouse anti-FMRP (1:1000; Developmental Studies Hybridoma Bank, catalog #2F5-1-s, RRID: AB_10805421) and POMC or MC4R antibodies as above. After PBST washes, sections were incubated overnight at 4 °C with secondary antibodies: goat anti-rabbit IgG-Alexa Fluor 488 (1:5000, Invitrogen, A11034); anti-mouse biotin (1:1000, BA-9200, Vector Laboratories, Burlingame, CA) followed by streptavidin-Cy5 (1:1000, 434316, Vector Laboratories, Burlingame, CA). Slices were mounted on slides with Vectashield mounting medium containing DAPI (Vector Laboratories, H-1200).

To determine puncta density, we followed our established protocol as previously published^19,36,37^. Puncta were counted in the individual neurons using z-stacks acquired by confocal Leica SP2 microscope. Images were encoded for blind analysis. At least 15–20 individual neurons from three different sets of mice were counted. 3D reconstruction was performed by Imaris software (Bitplane, Inc; Concord, MA).

### Western blot

Whole cell lysates were obtained from the dissected hypothalami from WT controls and FMR1 knockout mice and after protein determination, the same amount of protein from each sample was resolved on SDS-PAGE, transferred on nitrocellulose membrane and probed for: GABAγ2 receptor subunit (1:1000, 14104-1-AP, Proteintech), VGAT (1:1000, 131 011, Synaptic Systems), MC4R (1:1000, ab24233, Abcam) or β-tubulin (1:1000, sc-9104, Santa Cruz Biotechnology) (Supplementary information). Bands were quantified using ChemiDoc imaging system (Bio-Rad, Hercules, CA).

### Statistical analyses

Statistical differences between WT control and *Fmr1* KO mice (p < 0.05) were determined by t-test, or ANOVA when appropriate, followed by Tukey’s post-hoc test for multiple comparisons using Prism software (GraphPad, CA).

### Statement of ethics

All experiments were performed with approval from the University of California (Riverside, CA) Animal Care and Use Committee and in accordance with the National Institutes of Health Animal care and Use Guidelines.

## Results

### *Fmr1* knockout male mice are heavier due to lower locomotor activity and olfactory impairment

**Figure 1:**
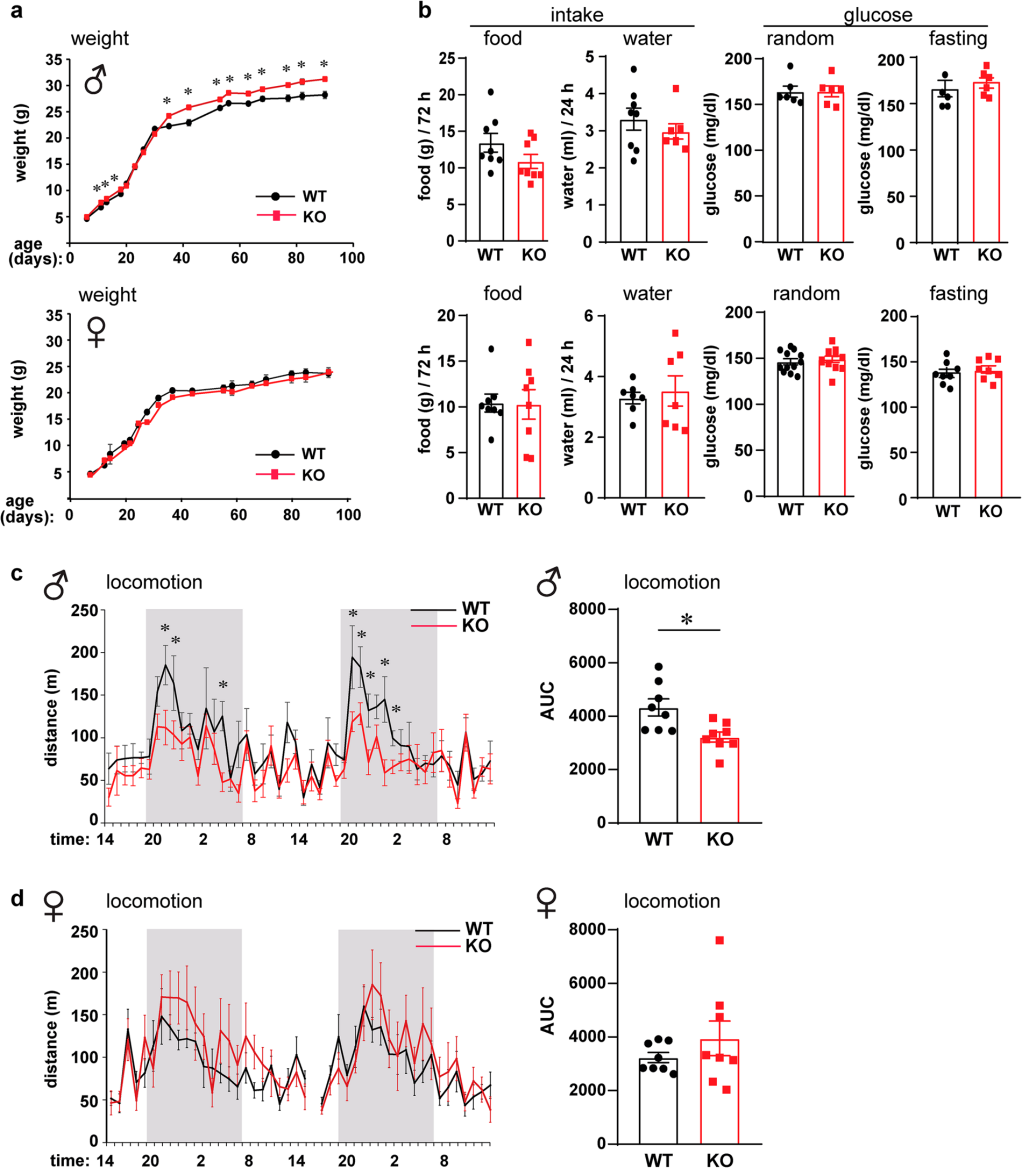
*Fmr1* knockout (KO) male mice are heavier than controls. (**a**) Litter sizes were normalized to 8 mice per litter, weight of 4 litters per genotype monitored from p7-p90, and *Fmr1* KO mice compared to wild-type FVB controls (WT) (KO, red; WT controls, black; males, top; females, bottom). n = 12–16 mice, points represent group mean ± standard error. Statistical significance between weight at each age is represented with * (p < 0.05, ANOVA followed by Tukey’s post hoc test). (**b**) Food (left) and water (right) intake were not different (cumulative, n = 8 per genotype); glucose levels (random, left; measured after overnight fasting, right) were not different (WT, black bars represent group mean ± standard error, each black circle represents one animal; KO, red bars represent group mean ± standard error, each red square represents one animal). (**c**) Locomotion activity of male mice measured continuously with Phenomaster for 48 h. Left, x-axis indicates time of day, when monitoring started at 14:00 or 2 pm, shaded area represents dark cycle, lights off in our vivarium from 7 pm to 7 am; black line, WT; red line, *Fmr1* KO; n = 8 mice, points represent group mean ± standard error; WT, black; KO, red. Right, area under the curve (AUC), WT, black bars represent group mean ± standard error, each black circle represents one animal; KO, red bars represent group mean ± standard error, each red square represents one animal. (**d**) Locomotion activity, female mice. Statistical significance (*, p < 0.05) was determined with t-test followed by Tukey’s post hoc test.

Children and adolescents with FXS are heavier than age-matched controls, but it is not clear if this is due to food preference or due to the effects of the *FMR1* gene mutation on the hypothalamic feeding circuitry. To address this question, we monitored the weight of *Fmr1* KO mice and compared them to WT controls. Given that *Fmr1* KO mice have larger litters^19^, we controlled for the number of pups per litter on the day of birth, since litter size affects early food intake and consequently weight. We normalized litter sizes to 8 pups per litter, and 4 different litters were analyzed. We monitored pups’ weight from postnatal day 7 (p7), when we can distinguish males and females, to p90; and determined that *Fmr1* KO male mice were heavier than WT controls (Fig. 1a, top). Female homozygous *Fmr1* KO mice did not show differences in weight (Fig. 1a, bottom), consistent with the observations that homozygous FXS females have less severe outcomes than males^38–41^. We determined that from p10, *Fmr1* KO male mice were heavier (p11 WT = 6.82 g, KO = 7.67 g, p = 0.012). The weight difference was lost for 10 days after weaning, from p21-p30, likely due to increased anxiety of *Fmr1* KO mice after separation from their mothers, that has been demonstrated^41,42^. At p35 and after, until p90, *Fmr1* KO male mice were again significantly heavier than WT mice (p42 for example, WT = 22.9 g, KO = 25.8 g, p = 0.00023). We then investigated if weight gain stems from increased food intake, and we individually housed WT and KO mice in two-hopper feeding chambers. After a three-day acclimation period, food intake and water consumption, and locomotor activity were measured in real time for another 3 days. Animals received ad libitum access to standard chow and water throughout testing. There was no difference in food or water intake, in males or females KO mice compared to controls (Fig. 1b. left). To determine if there are differences in blood glucose levels, we measured circulating glucose at p42, at random and after a 12-h fast, but did not detect any differences in either males or females (Fig. 1b, right). Using Phenomaster chambers to monitor locomotor activity, we determined that there was a decrease in movement of *Fmr*1 KO male mice compared to WT (Fig. 1c). This decrease was significant during the dark cycle, which is consistent with previous reports^43^. WT controls exhibited increased locomotion at the onset of the dark phase, when mice normally experience their active period and initiate foraging for food. *Fmr1* KO males exhibited smaller increase in this circadian locomotive activity. Area under the curve (AUC) was calculated to demonstrate overall significantly reduced locomotion of male mice, WT = 4330 compared to KO = 3223 (p = 0.015; Fig. 1c, right). Females did not show a significant difference in locomotion, and in fact, KO females exhibited a trend of moving more, which may correspond to hyperactivity^44^. AUC for female mice were WT = 3234 compared to KO = 3949 (Fig. 1d, right). Therefore, *Fmr1* KO males exhibit increased weight, no difference in food intake, and reduced locomotor activity compared to controls, which likely contributes to increased weight^45^.

**Figure 2:**
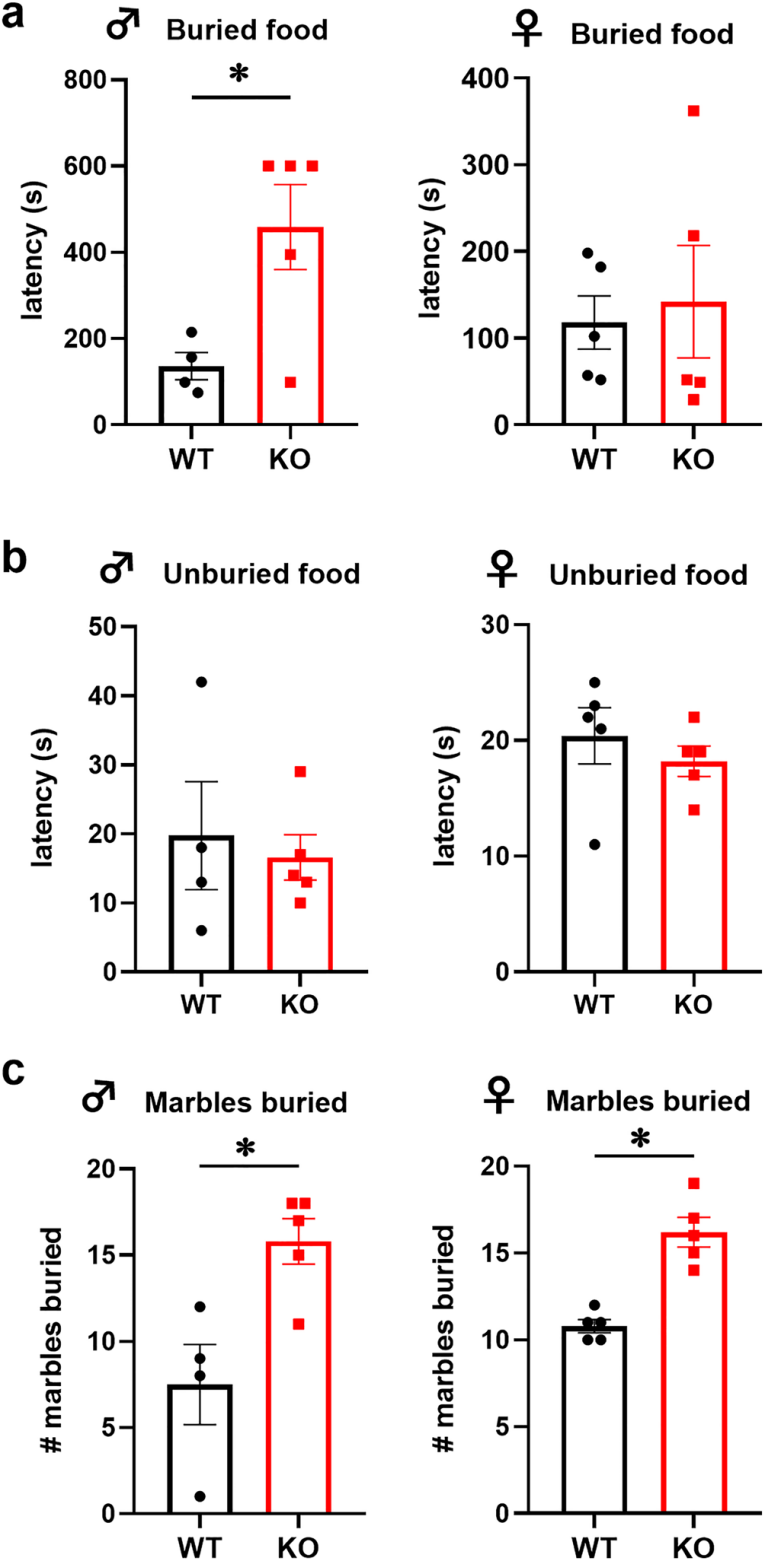
Buried food test demonstrates olfactory impairment in *Fmr1* KO male mice. (**a**) Left, WT male mice reached buried food pellet faster than KO mice (p = 0.0263; WT, black bars represent group mean ± standard error, each black circle represents one animal; KO, red bars represent group mean ± standard error, each red square represents one animal). Right, no difference in latency to uncover buried food pellet in female mice. (**b**) No difference in latency to retrieve unburied food pellet between KO and WT mice (left, male; right, female). (**c**) Left, WT males buried fewer marbles than KO male mice (p = 0.0135). Right, WT females buried fewer marbles than KO females (p = 0.0004). Statistical significance, indicated with * was determined with t-test followed by Tukey’s post hoc test.

Given that mice increase locomotion at the beginning of their active period, in order to reach the food pellets and eat, we investigated if *Fmr1* KO mice exhibit decreased locomotion due to the inability to smell food. The buried food test measures the latency to uncover a small piece of chow and is a reliable method to assess olfaction, since it uses animals’ natural propensity to use olfactory cues when foraging for food^46^. We determined that *Fmr1* KO male mice showed significant delay in reaching the food pellet. WT males uncovered the food pellet at 136.5 s, while KO reached the pellet at 458.8 s (Fig. 2a, p = 0.0263). There was no difference in latency of female mice to reach the buried food pellet, which may be consistent with lack of differences in locomotion.

As a control to assess motivation to eat, we performed an unburied food test, where the food pellet was left on the top of the bedding, after which the mouse was introduced to the cage in the opposite corner. There was no difference in the latency to reach the pellet and start eating between KO and WT mice when the food is visible, in male or female mice (Fig. 2b). We concluded that KO mice experience hunger signals, consistent with food intake analyses reported in Fig. 1b. Given that KO mice exhibited a delay in digging for food, we wanted to determine whether *Fmr1* KO mice have aversion to digging and performed a marble burying test^35^. Although *FMR1* mutations are associated with autism-spectrum disorders, which is characterized by repetitive or compulsive behaviors, we considered the marble burying test adequate to control for the willingness to dig, since these are natural and spontaneous behaviors in mice. We determined that *Fmr1* KO male and female mice buried significantly more marbles (Fig. 2c). WT male mice buried 7.5 marbles out of 20 during the 30-min test period, while KO male mice buried 15.8 marbles (Fig. 2c, left; p = 0.0135). WT female mice buried 10.8 marbles, compared to KO female mice that buried 16.2 marbles (Fig. 2c, right; p = 0.0004). These results demonstrate that *Fmr1* KO mice exhibit increased repetitive behaviors consistent with models of autism. Significantly for our studies, *Fmr1* KO male mice have an affinity to dig and therefore, delay in obtaining a food pellet stems from reduced olfaction.

### *Fmr1* gene deletion results in increased GABAergic innervation, lower activity, and fewer POMC neurons

**Figure 3:**
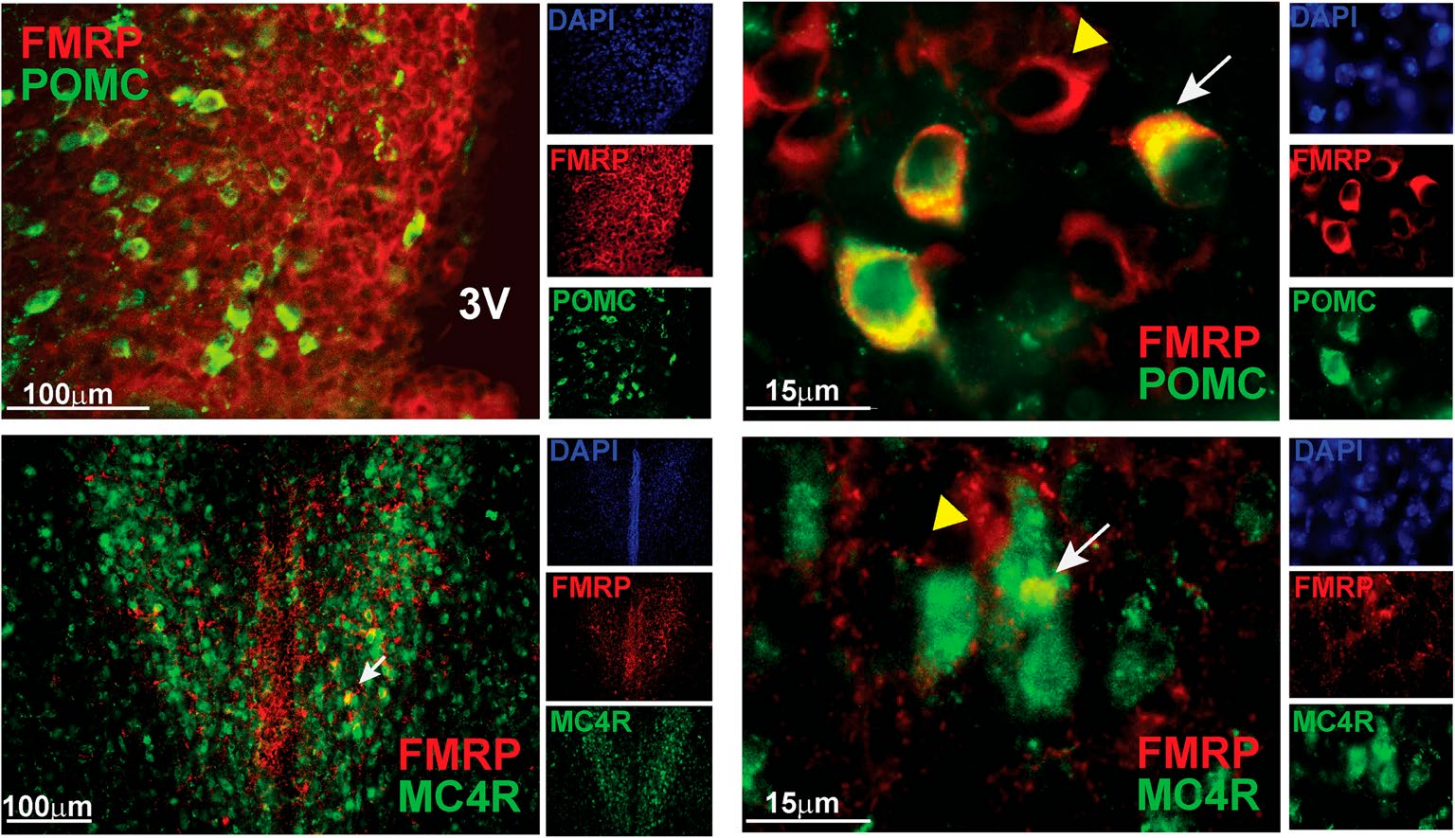
POMC neurons express FMRP. Top, POMC neurons in arcuate nucleus stained with anti-β-endorphin (red) and anti-FMRP (green). Representative images using ×20  objective (3 V, third ventricle, left); and ×63 objective (right). Bottom, MC4R neurons in the PVN stained with anti-MC4R (red) and FMRP (green). DAPI was included to detect the nuclei (separate channels on the side), but omitted in overlay to better visualize co-localization of FMRP with neurons of interest.

To answer if olfactory impairment in males regulates locomotion via feeding circuitry in the hypothalamus, we examined POMC neurons in male mice, since POMC neurons regulate energy expenditure^47,48^, and ablation of POMC neurons reduces locomotion in the dark phase^21^. POMC neurons regulate energy expenditure by targeting MC4R neurons, primarily in the paraventricular nucleus (PVN) of the hypothalamus^49^. POMC neurons also target MC4R expressing neurons in the premotor network, that stimulate locomotion^50^. We determined that 84% of POMC neurons expressed FMRP, while only 9% of MC4R expressing neurons in the PVN contained FMRP (Fig. 3). Therefore, we concentrated on POMC neurons and examined their innervation.

**Figure 4:**
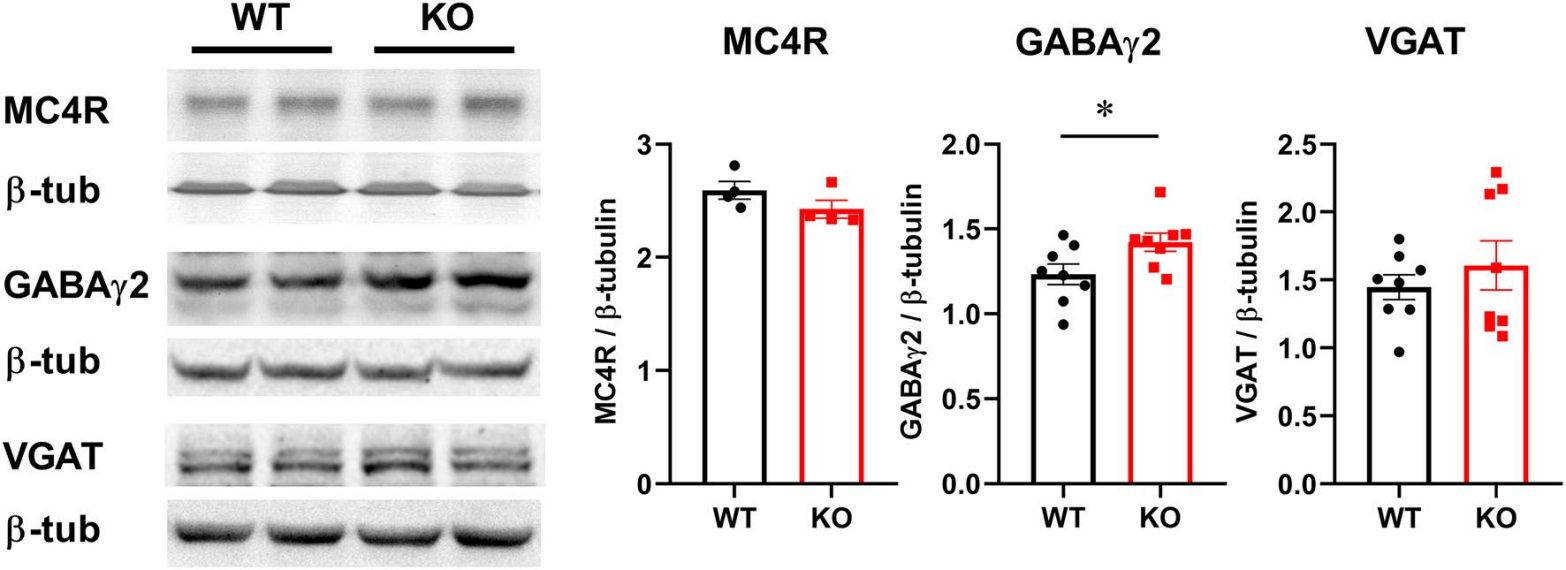
Western blots of the hypothalami whole cell lysates determined changes in synaptic proteins in *Fmr1* KO male mice. Hypothalami were dissected and protein levels were analyzed by western blotting. Protein levels of 4–8 mice per group were quantified using ChemiDoc and amount of the protein of interest normalized to the amount of β-tubulin. Each point represents one animal, while bars represent group means ± standard error. Statistical significance, indicated with a * (p < 0.05) was determined with t-test followed by Tukey’s post hoc test.

We then analyzed MC4R protein levels, as targets of POMC neurons via αMSH, and determined that MC4R levels are the same in WT and KO hypothalami, using western blotting (Fig. 4). Activation of POMC neurons occurs via their disinhibition through reduced GABA innervation. Hence, we examined levels of GABA_A_ receptors and VGAT in the hypothalami of *Fmr1* KO compared to WT. GABARγ2 is the obligatory subunit of the pentameric GABA_A_ receptor^51^, while vesicular GABA transporter (VGAT), is a presynaptic marker of GABAergic terminals^52,53^. There was a significant increase in the levels of GABARγ2 protein in the hypothalami of *Fmr1* KO male mice, consistent with our previous observations in female mice^19^. However, VGAT levels were not different (Fig. 4).

**Figure 5:**
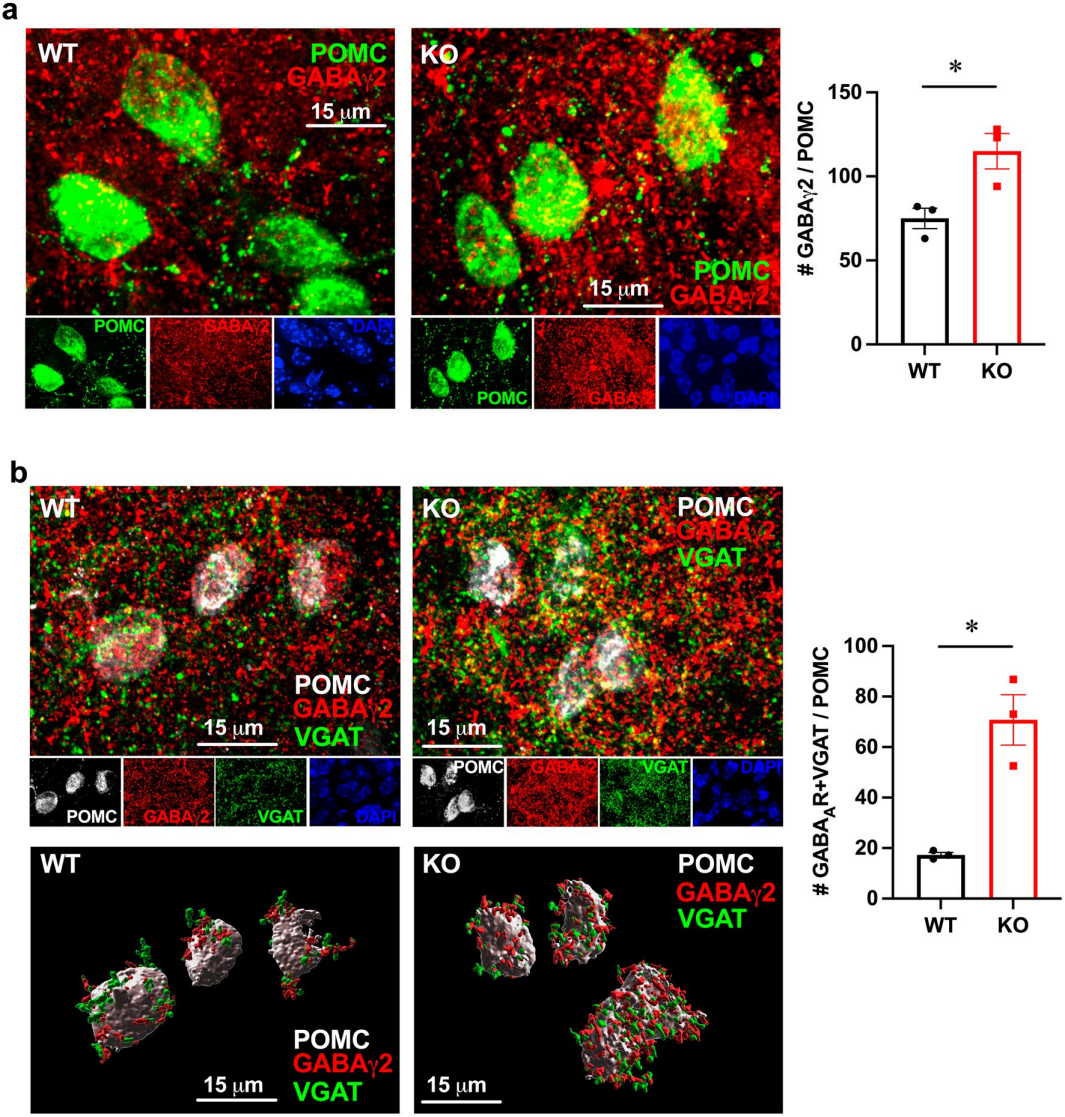
*Fmr1* KO males have increased GABAergic innervation. (**a**) GABA_A_ receptors in the POMC neuron soma were quantified; (**b**) synaptic GABA_A_ receptors were quantified as appositions of GABAγ2 and VGAT in POMC neurons. GABA_A_ (red), VGAT (green), POMC (grey). At least 20 neurons per mouse were quantified and the average for each mouse was represented with a point, while bars present genotype average. Bottom, 3D reconstruction of confocal images using Imaris. Statistical significance, indicated with a * (p < 0.05) was determined with t-test followed by Tukey’s post hoc test.

We next analyzed the GABAergic innervation of POMC neurons. First, we counted the number of GABA_A_ receptors in the POMC neuron somata and determined a significant increase in *Fmr1* KO mice to 154% from WT (Fig. 5a). To determine the number of synaptic GABA_A_ receptors we performed triple stain for VGAT, GABA_A_ and POMC, since VGAT is located in GABAergic presynaptic terminal. We stained for β-endorphin to detect POMC neurons, GABAγ2 and VGAT and counted the number of puncta where VGAT was located in a close opposition to GABAγ2 in POMC neurons. At least 15 neurons per mouse, 3 mice per group, were counted (Fig. 5b). We determined more than a fourfold increase (to 409%) in GABAergic innervation of POMC neurons in *Fmr1* KO mice.

**Figure 6:**
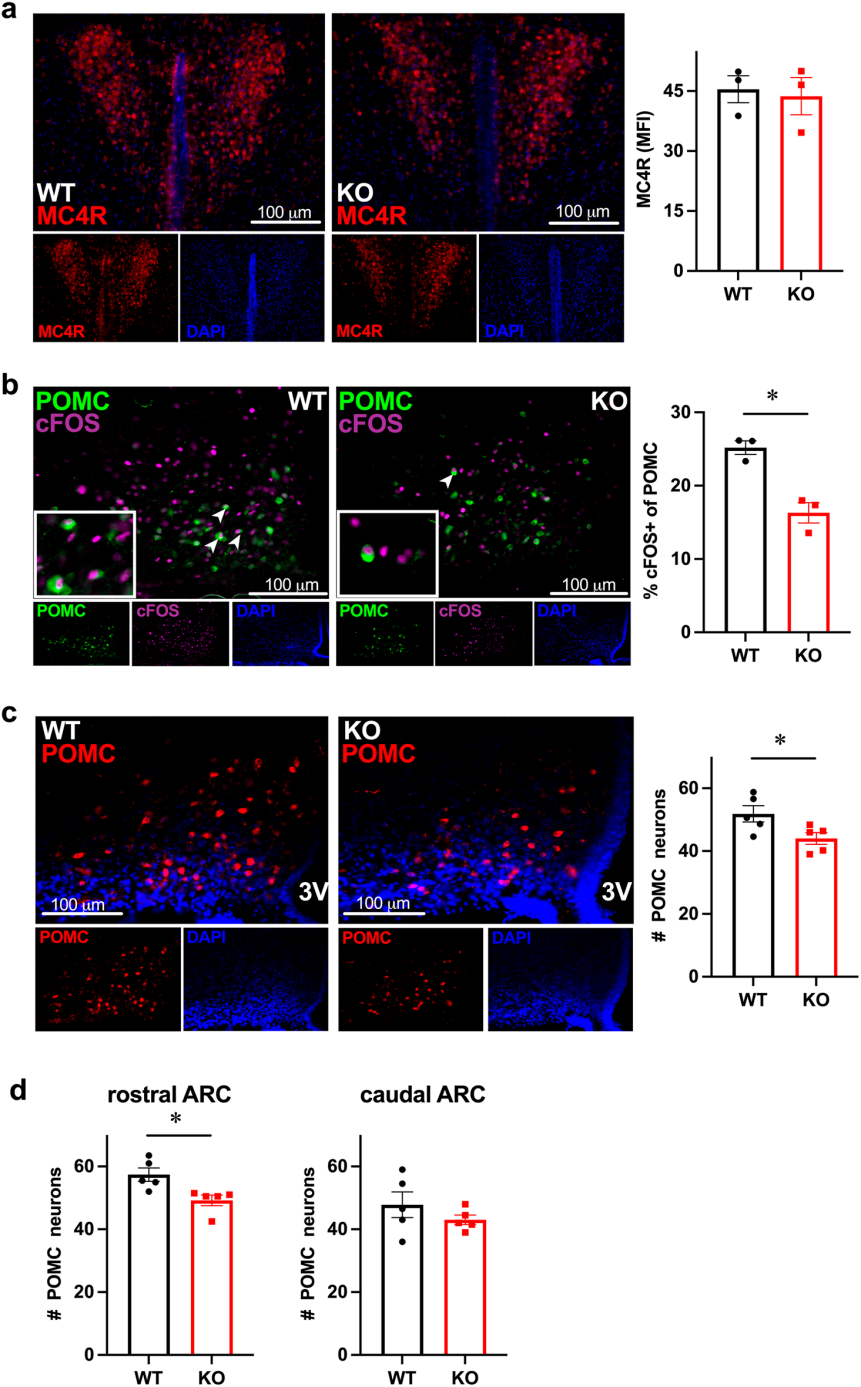
Decreased activity and numbers of POMC neurons in the arcuate nucleus of the hypothalamus in *Fmr1* KO male mice. (**a**) No difference in the MC4R neuron number in the PVN between WT and KO male mice, quantified as MFI using ImageJ; MFIs of 5–7 coronal sections per mouse were averaged; each dot represents one animal, bar represents group mean ± SEM. (**b**) KO males have fewer active POMC neurons compared to WT, determined by co-staining of POMC (green, cytoplasmic) and cFOS (magenta, nuclear). cFOS-positive POMC neurons are indicated with arrowheads, inserts contain higher magnification of double positive neurons. Right, numbers of cFOS positive POMC, shown as percentage. (**c**) KO males have fewer POMC neurons, determined by counting β-endorphin/POMC cell bodies throughout the arcuate nucleus in at least 6 sections per mouse and averaged; each dot represents one animal, bar represents group mean ± SEM. (**d**) Counts for the rostral and caudal regions demonstrated separately to indicate regional heterogeneity. Statistical significance, indicated with a * (p < 0.05) was determined with t-test followed by Tukey’s post hoc test.

To determine functional significance of changes in innervation, we counted the numbers of MC4R neurons in the PVN and POMC neurons throughout ARC in *Fmr1* KO and WT mice (Fig. 6). We determined that there was no change in the number of MC4R neurons in the PVN (Fig. 6a). We then assessed the activity of POMC neurons by staining for cFOS and counting the percentage of cFOS-positive POMC neurons (Fig. 6b). cFOS is located in the nucleus (magenta), while β-endorphin is cytoplasmic (green). *Fmr1* KO mice had significantly lower fraction of cFOS-positive POMC neurons of 16.3% than WT mice, 25.2% (Fig. 6b, p = 0.006; arrowheads indicate cFOS-positive neurons; inset, higher magnification of these positive neurons indicated by arrows). *Fmr1* KO mice also had 15.2% fewer POMC neurons in the arcuate nucleus of the hypothalamus (Fig. 6c, p = 0.04). Given the functional and regional heterogeneity of POMC neurons, we counted POMC neurons separately in the rostral region of the arcuate nucleus and in the caudal region. We determined that rostral region specifically had 24% fewer POMC neurons in *Fmr1* KO than WT controls, while caudal region lacked significant difference (Fig. 6d, p = 0.016). Given that POMC neurons regulate locomotion via αMSH activation of MC4R-neurons in the premotor area^54,55^, we postulate that this decrease in POMC neuron number causes decreased energy expenditure due to lower innervation of premotor area, which leads to increased weight gain.

## Discussion

We sought to uncover the effects of the loss of FMRP on hypothalamic neurons and energy expenditure, which may help elucidate the mechanisms of weight gain in people affected by FXS due to a mutation in the *FMR1* gene. Additionally, our studies uncovered a significant factor, the *FMR1* gene, in weight homeostasis and regulation of feeding circuitry. Our studies determined increased weight without increased food intake in *Fmr1* KO mice. We further determined that the weight gain, specifically in male mice, may be due to lower locomotion during the dark cycle, which in turn may be due to impaired olfaction and the inability to smell food. These results point to the critical role of FMRP in the olfactory-feeding circuitry axis. Furthermore, we determined that POMC neurons that regulate energy expenditure receive higher GABAergic innervation and exhibit lower activity and fewer numbers in *Fmr1* KO mice. Therefore, we posit that POMC neurons are the linchpin between olfaction, hunger signals, and locomotion when foraging for food.

Our results may explain reasons for the increased prevalence of obesity in people affected with FXS. Body weight increase of *Fmr1* KO mice to 115% of controls is similar to what was observed in mice with a deletion of leptin receptor specifically in POMC neurons causes, which experienced 120% weight gain compared to controls^56^. The increase in body weight in *Fmr1* KO mice of the FVB background that we observed here, is consistent with an increase in body weight of *Fmr1* KO mice on C57 background reported before^57^. Report by Leboucher, et al., postulated that the increase in body weight is due to the changes in body composition, specifically an increase in muscle mass and bone volume, while we provide additional possibilities, such as decreased locomotion or POMC neuron dysfunction that may contribute to weight gain. An earlier study by the same group analyzed metabolic indicators in *Fmr1* KO mice^58^ and found a lack of differences in glucose levels, which is in agreement with our study, but reported differences in serum lipid profile, leptin and insulin. Our study may be in agreement with a study using a zebrafish model of *Fmr1* deletion, that determined developmental defects in the motor cortex, which may lead to changes in locomotion^59^. Also in agreement with our results here, decreased locomotion in the *Fmr1* KO mice on the C57 background was reported before, in a report that demonstrated that reduced movement is due to reduced forward locomotion and bout onsets^43^. This study also did not find any differences in food intake similar to our results, but contrary to our study and studies by Lebousher, et al., did not find differences in weight or body composition either. Another study using slightly older mice, did not detect differences in weight^60^, may be because of differences in age. Alternatively, since *Fmr1* KO mice have larger litters^19^, we normalized the litter size, which may explain different results concerning body weight.

We detected an increase in GABA_A_ receptor levels in the hypothalami of *Fmr1* KO mice. A number of studies determined a decrease in several GABA_A_ receptor subunits, in the cortex and hippocampus of KO mice, implicating a decrease in inhibitory signaling in the neuropathology of the disease^61–64^. Indeed, *Fmr1* KO mice show cortical hyperexcitability similar to FXS individuals^65,66^. However, these differences in GABA_A_ receptor levels were brain region specific and were not observed in the cerebellum^64,67^. We show increased GABA_A_ receptor levels in the hypothalami of *Fmr1* KO mice, indicating additional regional differences. GABA is the dominant neurotransmitter in the hypothalamus^68^ and GABAergic neurons are first order neurons that receive information from periphery^69^, indicating the importance of inhibitory circuits in the hypothalamus. Based on findings of decreased GABA_A_ receptor levels in several brain areas, GABA_A_ receptor agonists were proposed as possible pharmacological treatments for FXS symptoms^70–72^. Given our results, this pharmacological approach may increase incidence of obesity in individuals affected by FXS. Of note, GABA_A_ receptor agonists cause weight gain and a decrease in locomotor activity when administered to rats^73^.

We detected lower activity and fewer POMC neurons in *Fmr1* KO mice. POMC neurons regulate locomotion and energy expenditure. An increase in energy expenditure occurs via αMSH binding to the MC4R receptors^21^. However, mechanisms whereby POMC neurons regulate locomotion are less clear. Animals increase locomotion to locate food and POMC neurons may be involved in this anticipatory behavior^48^. POMC neurons directly innervate spinal cord regions of the premotor networks via long-range projections to MC4R-expressing V2a interneurons^50^. Furthermore, leptin via leptin receptor expression exclusively in POMC neurons of leptin receptor KO mice is sufficient to stimulate locomotion^74^. AgRP neurons may also be involved, since DREADD-mediated acute activation of AgRP neurons decreased locomotor activity during the dark cycle^75^. Due to the inability to examine AgRP neurons in *Fmr1* KO mice, without crossing to an AgRP-reporter strain, we examined POMC neurons, since AgRP neurons regulate feeding and satiety, in part, via GABAergic innervation of POMC neurons^23,24^. We determined a decrease in the activity and number of POMC neurons, which may contribute to decreased locomotion.

POMC neurons are a heterogeneous population^47,48^. In addition to the population in the arcuate nucleus of the hypothalamus with about 5000 neurons, there is a small population of about 200 POMC neurons in the nucleus of solitary tract (NTS), whose function is not clear. Diphtheria toxin–mediated ablation of POMC neurons in the ARC but not the NTS, increased food intake, reduced energy expenditure, and decreased locomotor activity in the dark stage^21^. For that reason, we focused on POMC neurons in the arcuate nucleus. However, there is further functional and regional heterogeneity within the hypothalamic POMC neurons. Addressing regional heterogeneity, rostral arcuate nucleus POMC neurons project mostly to autonomic areas in the brainstem, such as the dorsal vagal complex^76,77^. POMC neurons in the caudal part of the arcuate nucleus project to hypothalamic areas, including the PVH. Due to this regional heterogeneity, we counted POMC neurons in the rostral and caudal portions of the arcuate nucleus, separately, and detected a decrease in the rostral POMC population. Findings that POMC neurons from the rostral part of the arcuate nucleus project to the brainstem, which controls locomotion^54,55^, may correspond to our results or decreased locomotion and fewer rostral POMC neurons in *Fmr1* KO male mice. Functional heterogeneity of POMC neurons within the arcuate nucleus may indicate subpopulations with different roles in the regulation of energy balance, locomotion, food intake, glucose metabolism, and lipid levels^48^. Further studies are needed to explain if differences in connectivity, neurotransmitter and neuropeptide receptor composition cause the functional heterogeneity.

Foraging for food is guided by olfactory cues and is accompanied by increased locomotion. *FMR1* is expressed throughout the brain regions associated with olfaction^78^. *Fmr1* KO mice exhibit olfactory dysfunctions and decreased ability to detect an odorant, but lack differences in the ability to habituate to odorants or to discriminate between them^29^. Study in flies determined that FMRP expression is required to process olfactory information and create context-dependent memories^79^. Structural and morphological changes in olfactory brain regions have also been reported in *Fmr1* KO mice^13,78^. Our results using food pellet detection in the buried food test and marble burying test identified an olfactory deficit. The buried food-seeking test, which tests whether the food-deprived mice can find the food pellet hidden beneath the bedding, determined that KO male mice exhibit a delay, likely due to inability to smell food. Controls for motivation to eat, such as unburied food test, and for burying ability, such as marble burying test, determined that KO mice have the same motivation to eat as WT, consistent with lack of differences in food intake; and that KO mice buried more and thus, exhibit increased repetitive behavior, consistent with *Fmr1*-associated compulsive disorders. Important for our studies, this control determined that KO mice have the ability to dig in the bedding. Although it is clear that smelling food increases appetite, the connections between olfactory circuits and feeding circuits are not completely elucidated. Several studies demonstrated the connectivity between olfactory brain regions and the hypothalamic regions that regulate food intake^80^. Specifically, connections between the olfactory bulb and the hypothalamic arcuate nucleus where POMC neurons are located have been described^80–82^. Sensory detection of food after pellet presentation to mice activated POMC neurons^83^, indicating that POMC neurons may be involved in anticipatory behavior in preparation for food intake^48^.

Therefore, it is possible that olfactory dysfunction dysregulates POMC neurons, which in turn decrease locomotion in the form of food seeking behavior. This decrease in locomotion reduces energy expenditure and contributes to weight gain, despite the lack of differences in overall food intake.

### Supplementary Information

Supplementary Information.

## Data Availability

The datasets generated during and/or analyzed during the current study are available from the corresponding author on reasonable request.
