# Supplementary material for: Increased body weight in mice with fragile X messenger ribonucleoprotein 1 (Fmr1) gene mutation is associated with hypothalamic dysfunction
Source: Sci Rep. 2023 Aug 4;13:12666. doi: 10.1038/s41598-023-39643-z (PMC10403586; doi:10.1038/s41598-023-39643-z)
Supplement: Supplementary file 1 — Supplementary Information. [file 41598_2023_39643_MOESM1_ESM.pdf]

Western blots included in Fig. 4

indicates area presented in Fig. 4

indicates protein of interest based on molecular weight (mw)

anti-GABAγ2

mw wt0 wt1 wt2 wt3 ko1 ko2 ko3 ko0

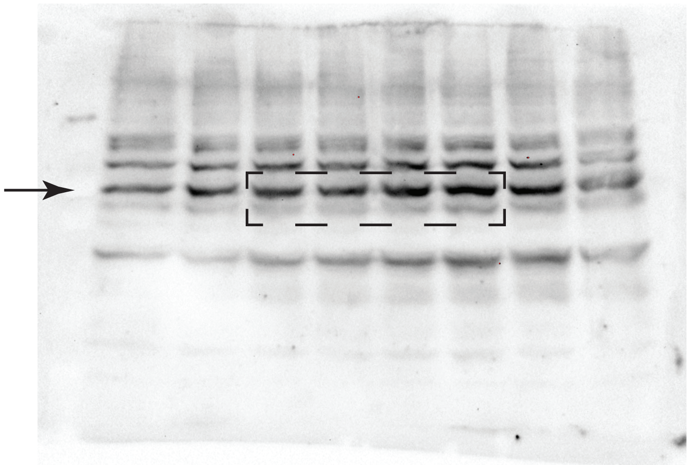

anti-β-Tubulin

mw wt0 wt1 wt2 wt3 ko1 ko2 ko3 ko0

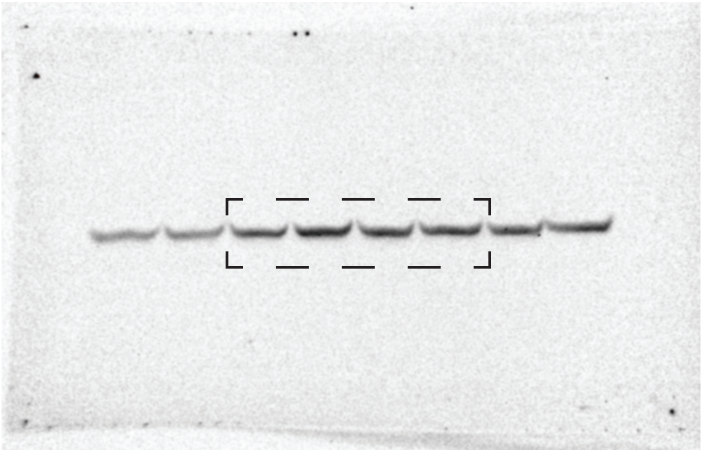

anti-VGAT

mw wt0 wt1 wt2 wt3 ko1 ko2 ko3 ko0

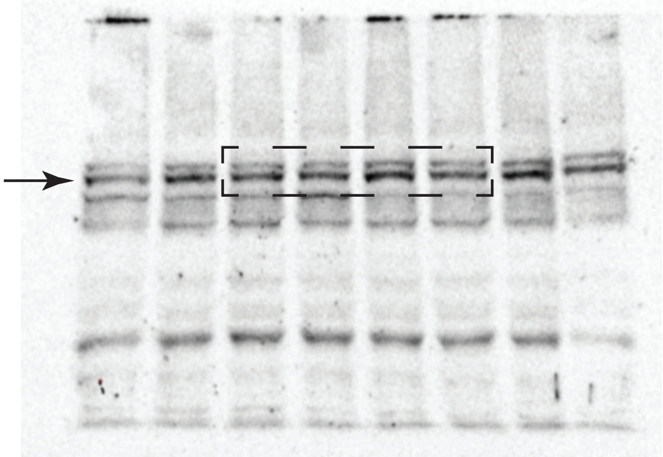

anti-β-Tubulin

mw wt0 wt1 wt2 wt3 ko1 ko2 ko3 ko0

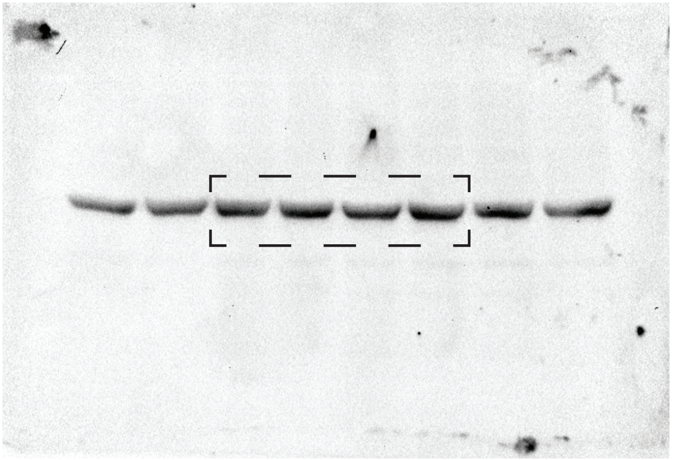

anti-MC4R

wt0 wt1 wt2 wt3 ko1 ko2 ko3 ko0 mw

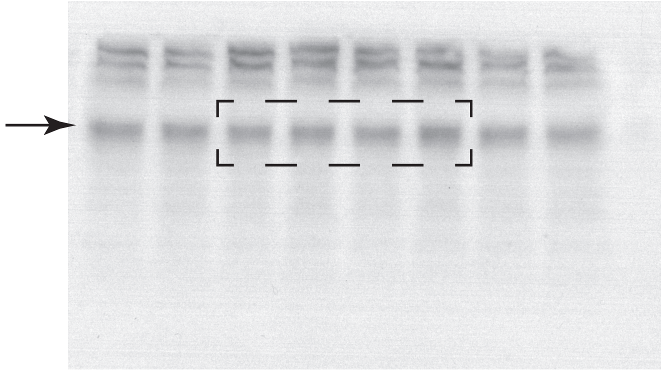

anti-β-Tubulin

wt0 wt1 wt2 wt3 ko1 ko2 ko3 ko0 mw

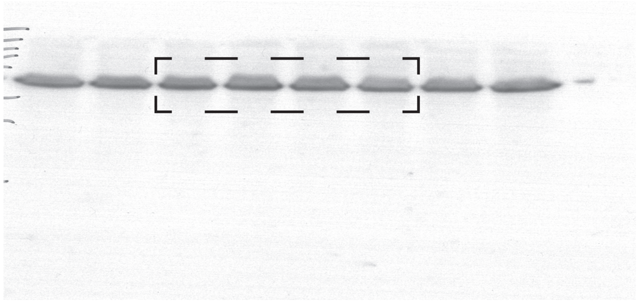

Supplementary Figure 1
